# Design of experiments to assess the effect of culture parameters on the osteogenic differentiation of human adipose stromal cells

**DOI:** 10.1186/s13287-019-1333-7

**Published:** 2019-08-14

**Authors:** Mirasbek Kuterbekov, Paul Machillot, Francis Baillet, Alain M. Jonas, Karine Glinel, Catherine Picart

**Affiliations:** 1 Université catholique de Louvain, Institute of Condensed Matter & Nanosciences (Bio & Soft Matter), Croix du Sud 1, Box L7.04.02, 1348 Louvain-la-Neuve, Belgium; 2 CNRS, LMGP, 3 parvis Louis Néel, 38016 Grenoble, France; 3 Grenoble Institute of Technology, University Grenoble Alpes, LMGP, 3 parvis Louis Néel, 38016 Grenoble, France; 4 Université Grenoble Alpes, CNRS, Grenoble INP, SIMAP, 1130 rue de la Piscine, 38402 Saint-Martin d’Hères, France

**Keywords:** Human adipose stromal cell, Osteogenic differentiation, Design of experiments, Cell therapy

## Abstract

**Background:**

Human adipose-derived stromal cells (hASCs) have been gaining increasing popularity in regenerative medicine thanks to their multipotency, ease of collection, and efficient culture. Similarly to other stromal cells, their function is particularly sensitive to the culture conditions, including the composition of the culture medium. Given the large number of parameters that can play a role in their specification, the rapid assessment would be beneficial to allow the optimization of their culture parameters.

**Method:**

Herein we used the design of experiments (DOE) method to rapidly screen the influence and relevance of several culture parameters on the osteogenic differentiation of hASCs. Specifically, seven cell culture parameters were selected for this study based on a literature review. These parameters included the source of hASCs (the different providers having different methods for processing the cells prior to their external use), the source of serum (fetal bovine serum vs. human platelet lysate), and several soluble osteoinductive factors, including dexamethasone and a potent growth factor, the bone morphogenetic protein-9 (BMP-9). The expression of alkaline phosphatase was quantified as a readout for the osteogenic differentiation of hASCs.

**Results:**

The DOE analysis enabled to classify the seven studied parameters according to their relative influence on the osteogenic differentiation of hASCs. Notably, the source of serum was found to have a major effect on the osteogenic differentiation of hASCs as well as their origin (different providers) and the presence of L-ascorbate-2-phosphate and BMP-9.

**Conclusion:**

The DOE-based screening is a valuable approach for the classification of the impact of several cell culture parameters on the osteogenic differentiation of hASCs.

**Electronic supplementary material:**

The online version of this article (10.1186/s13287-019-1333-7) contains supplementary material, which is available to authorized users.

## Background

Human adipose-derived stromal cells (hASCs) are an attractive candidate for a large variety of cell-based therapies in regenerative medicine [1]. One of their most promising applications is in the regeneration of bone tissues [2]. This application stems from their ability to undergo osteogenic differentiation, in which they exhibit osteoblast-like function leading to the deposition of an extracellular matrix with its subsequent mineralization [3]. Accumulating research has shown that hASCs, like many other mesenchymal stromal cells, are highly sensitive to culture conditions which affect their various cell functions, including osteogenic differentiation [1]. This is particularly important as researchers are paying increasingly close attention to the origins of biochemicals used in order to comply with Good Manufacturing Practices (GMP) and facilitate clinical translation. For this reason, significant efforts are being dedicated to replacing xenogeneic cell culture products with their allogeneic counterparts. One example is the replacement of fetal bovine serum (FBS) with human platelet lysate (hPL) [4]. Given the sensitivity of the cells to the culture conditions, such replacements must be studied in detail to ensure that they do not alter cellular functions. Significant advantages conferred on hASCs by the use of hPL, e.g., increased proliferative potential [5] and improved chromosomal stability [6], have already led to its use as the serum supplement, including in our previous study [7]. However, some reports have indicated that hPL might lead to the spontaneous expression of alkaline phosphatase (ALP), an important marker of the osteogenic differentiation [8]. This induction occurred in the absence of any other osteoinductive supplementation, resulting in compromised negative controls.

In order to elucidate the effect of hPL on the osteogenic differentiation of hASCs, in addition to other culture parameters such as the source of stem cells (different providers having different processing methods), seeding density, and various medium components, we applied the concept of design of experiments (DOE). This statistical approach allows to describe and model the variation of a set of readouts based on input variables. One of its advantages is the ability to provide descriptive assessments using the minimally required number of experimental conditions [9]. Importantly, by targeting several variables at a time, DOE can help in identifying important interactions that may have been missed when analyzing these variables separately. Besides, the methodology provided by DOE ensures that the experiments are made in a statistically equilibrated manner within the selected working domains of the variable parameters. After specifying the range of variability for each variable, which may be discrete or continuous, it generates an experimental matrix with the minimum number of experimental conditions to be analyzed. Regularly used in chemical and mechanical engineering for process optimization and predictive modeling, DOE is still relatively uncommon in the fields of cell biology and bioengineering. Very few studies use DOE applied to biological questions. DOE was used to optimize the culture medium composition for the expansion of human pluripotent stem cells [10] and the design of hydrogel substrates for their neurogenic differentiation [11]. To the best of our knowledge, this approach has not yet been applied to analyze culture parameters for the osteogenic differentiation of mesenchymal stromal cells.

The goal of the present study was to apply the DOE approach to rank the aforementioned variables for their contribution to maximizing the osteogenic differentiation of hASCs. Given that the main interest was in ranking individual variables, a fractional factorial design, which omits intervariable interactions in order to further reduce the number of required conditions, was employed. One such design, known as Plackett-Burman design [12], uses a Hadamard matrix to define this minimal number of runs. This number is the minimal number of experimental conditions tested in order to determine a linear model without interaction between the variables. The aim of this model is to assess the impact of the different variable parameters on the outcome (e.g., measured biochemical signal) and to verify that the variation induced by a single parameter is larger than the experimental uncertainty.

As negative and positive controls are essential for an accurate analysis, extra conditions corresponding to such controls were added to the matrix. To allow for the rapid assessment, the expression of ALP, a common early marker for osteogenic differentiation, was set as the sole target response for both analyses. It was analyzed using a standard colorimetric assay and quantified using absorbance detection [13].

## Methods

All reagents and products were purchased from Sigma-Aldrich and Thermo Fisher Scientific unless stated otherwise.

### Selection of hASC culture parameters

Following a literature review, the focus was given to eight variables that are considered to wield the most influence on the osteogenic differentiation of hASCs (Table 1). The first of these is the source of stem cells as several publications have shown that hASCs comprise heterogeneous populations with differential capacities for osteogenic differentiation [14]. To this end, we used hASCs supplied by Zen-Bio (hASC-ZB), Inc. and Établissement Français du Sang (hASC-EFS). The surface markers reported for both types of cells are described in Additional file 1: Section 1. The second variable is the seeding density as it has been shown to affect the proliferative capacity of hASCs [5]. Given the importance of culture medium on resulting hASC function, both the base medium and the serum [8] were chosen as the other variables. Particularly, we focused on Dulbecco’s modified Eagle’s medium (DMEM) with and without Ham’s F-12 for the base culture medium and xenogeneic FBS and allogeneic hPL for the serum. As hASCs normally require supplementation [15] to undergo osteogenic differentiation, most frequently with dexamethasone, l-ascorbate-2-phosphate, and ß-glycerophosphate [16], they were included in the list of variables as well. Finally, bone morphogenetic proteins (BMPs) represent an important class of osteoinductive growth factors to drive the osteogenic differentiation of hASCs [17]. While BMP-2 and BMP-7 are commonly used for this purpose, a recent study has shown that BMP-9 may be more osteoinductive toward hASCs [18]. Thus, including BMP-9 as one of the variables might help in further elucidating its effect on the osteogenic differentiation of hASCs.

**Table 1:** Target variables used for the screening of culture parameters for the osteogenic differentiation of hASCs. hASC source and seeding density were chosen as factors related to the stromal cells; base medium, serum, L-ascorbate-2-phosphate, ß-glycerophosphate, dexamethasone, and BMP-9 were chosen as factors related to the medium. The factor variability was set between a certain range based on the values commonly used in literature

Variable	Description	Range of variability from (-) to (+)			References
V1	hASC source	ZB	EFS		[14]
V2	Cell seeding density, per well	2000	6000	10000	[5]
V3	Base culture medium	DMEM/F-12	DMEM		[15]
V4	Serum source	5% hPL	10% FBS		[8]
V5	L-ascorbate-2-phosphate	0	50 μM		[16]
V6	ß-glycerophosphate	0	10 mM		[16]
V7	Dexamethasone	0	100 nM		[16]
V8	Bone morphogenetic protein-9	0	100 ng/mL		[18]

### Construction of the experimental matrix and the DOE analysis

The 12 × 12 Hadamard matrix was used to accommodate the eight target variables, which allows to reduce the total number of conditions from 256 (2^8^, full factorial design) to 12 (fractional factorial design) [19]. This number of conditions is sufficient to obtain the coefficients of the linear model without interaction between the variables, in order to assess the impact of the different variables and to rank them according to their impact. Four additional conditions were added to the matrix to include the negative and positive controls for each of the hASC source. The resulting experimental matrix contained 16 conditions (Table 2**)** in total with an assigned value for each of the eight variables. The range of variabilities, particularly for numerical variables such as the seeding density or the concentrations of medium components, were approximated to those most commonly used in literature. Two types of variables were used in the construction of the experimental matrix: discreet variables such as hASC source, base culture medium, and serum source and continuous variables such as different medium supplements and osteoinductive factors, whose concentration can be controlled in a continuous fashion.

**Table 2:** The parameters used to generate the experimental table. In total, 16 conditions were investigated to screen culture parameters for their effect on osteogenic differentiation. *hASC* human adipose-derived stromal cell, *AP* l-ascorbate-2-phospate, *ßGP* ß-glycerophosphate, *DEX* dexamethasone, *BMP-9* bone morphogenetic protein-9. ^+^,^−^ refer to the four added positive and negative controls

Condition	hASC source	Seeding density (cells/well)	Medium compositions for osteogenic differentiation					
			Base medium	Serum	AP (μM)	ßGP (mM)	DEX (nM)	BMP-9 (ng/mL)
1	EFS	10,000	DMEM/F-12	hPL	50	10	0	0
2	ZB	10,000	DMEM	FBS	50	10	100	0
3	EFS	2000	DMEM	hPL	0	10	100	100
4	ZB	10,000	DMEM/F-12	hPL	50	0	100	100
5	ZB	2000	DMEM	FBS	0	10	0	100
6	ZB	2000	DMEM/F-12	hPL	0	10	100	0
7	EFS	2000	DMEM/F-12	FBS	50	0	100	100
8	EFS	10,000	DMEM/F-12	FBS	0	10	0	100
9	EFS	10,000	DMEM	FBS	0	0	100	0
10	ZB	10,000	DMEM	hPL	0	0	0	100
11	EFS	2000	DMEM	hPL	50	0	0	0
12	ZB	2000	DMEM/F-12	FBS	0	0	0	0
13^−^	ZB	6000	DMEM	FBS	0	0	0	0
14^+^	ZB	6000	DMEM	FBS	50	10	100	100
15^−^	EFS	6000	DMEM	FBS	0	0	0	0
16^+^	EFS	6000	DMEM	FBS	50	10	100	100

### hASC culture

Differentiation assays involved two steps: hASC expansion in a growth medium (GM) to obtain their sufficient numbers and the subsequent differentiation in a relevant osteogenic medium (OM). The aforementioned controls were used to confirm two things: the capacity of hASCs in question to undergo osteogenic differentiation (positive control, usually carried out through osteogenic induction in OM) and the fact that such differentiation is not spontaneous or self-induced (negative control, carried out by maintaining hASCs in GM).

#### hASC expansion and seeding

The influence of the cell source was studied by using hASCs from either Zen-Bio, Inc. (hASC-ZB) or the Établissement Français du Sang (hASC-EFS). Both cell lines were used before the 5th passage and cultured in FBS-based GM [DMEM + 10% FBS + 1% penicillin/streptomycin] with medium changes every 2 days. Upon seeding within 96-well cell-adherent microplates (Greiner Bio-One), hASCs were left undisturbed for a day to allow their attachment.

#### Pre-screening of the serum used during hASC expansion for the fidelity of negative and positive differentiation controls

Prior to launching the DOE analysis, the effect of FBS and hPL during hASC expansion on the ALP expression was analyzed to assess the fidelity of negative and positive controls. For this purpose, hASC-EFS were expanded in either FBS-based GM or hPL-based GM [DMEM + 5% hPL (Cook Regentec) + 1% penicillin/streptomycin]. 10,000 hASCs/well were seeded, followed by their osteogenic differentiation with the corresponding OM [GM + 100 nM dexamethasone + 50 μM L-ascorbate-2-phosphate + 10 mM ß-glycerophosphate]. hASCs expanded and maintained in FBS- and hPL-based GM served as negative controls.

#### hASC differentiation according to DOE conditions

hASC-EFS and hASC-ZB, expanded in FBS-based GM, were seeded at 2000, 6000, or 10,000 cells/well within the microplates with subsequent induction with the corresponding medium for osteogenic differentiation based on the conditions defined in Table 2. In total, 16 different medium formulations were prepared with the BMP-9 (PeproTech, 95% purity) added to the medium at the last minute to achieve the final concentration.

#### Analysis of the osteogenic differentiation of hASCs via ALP staining

The alkaline phosphatase (ALP) expression was analyzed after the osteogenic induction. In general, the analysis of ALP expression can be carried out through two complimentary methods, ALP staining [13] and ALP enzymatic activity [20]. Both methods which are colorimetric in nature were tested (Additional file 1: Section 2); however, the results of the analysis show that the ALP staining generated more reproducible readings as judged by the standard deviation (error bars) between technical replicates (Additional file 1: Figure S1). Moreover, ALP staining requires less sample manipulation and fewer steps than the enzymatic assay, which makes it attractive for the purposes of rapid and high-throughput analyses [13]. It is also much better adapted to the small working volumes of a 96-well cell culture microplate generating more reproducible readings (Additional file 1: Section 2). Therefore, ALP staining method was used to measure the ALP expression after osteogenic induction. Moreover, day 7 was chosen as ALP staining was sufficiently pronounced at this time point with cell layer detachment that was observed for later time points at 10 and 14 days (Additional file 1: Section 3 and Additional file 1: Figure S2). For the ALP staining, hASC-containing wells were washed twice with phosphate-buffered saline (PBS), fixed with formaldehyde (3.7% in PBS) for 20 min at room temperature and rinsed twice with PBS. The fixed cells were then incubated with the ALP staining solution (Leukocyte Alkaline Phosphatase kit, 120 μL/well) for 30 min at 37 °C and rinsed with PBS. Once dry, the images of the stained wells were taken with a scanner (Epson V600) and their absorbance at 570 nm was quantified by taking measurements over the entire area of each well at 11 × 11 positions using a microplate reader (TECAN Infinite M1000). Data were pooled from three biological experiments with three technical replicates for each experimental condition.

### Statistical analysis

Design-Expert® 11 (Stat-Ease), a versatile and commonly used software for DOE analyses, was used to encode the experimental table generated (Table 2) and statistically analyze the ALP staining results. Both discrete and continuous variables were encoded as such and the design parameters were set according to our chosen model (fractional factorial design, response modeling to the order of main effects only, ignoring second-order factor interactions). The output contained a table with numerical results for each variable, including their percent contributions to the observed readout. Microsoft Excel® was used to generate figures using these values. Numerical results from ALP staining are expressed as means ± standard deviation of the technical replicates. In the context of data collection, biological replicate refers to experiments performed on different days while technical replicates refer to concurrently run experiments within the same biological replicate. In terms of DOE results, if the (+) variability of the variable resulted in higher ALP expression, the contribution was deemed positive; if it led to lower ALP expression, the contribution was deemed negative.

## Results

### Effect of the serum used during hASC expansion on the fidelity of negative and positive differentiation controls

**Fig. 1:**
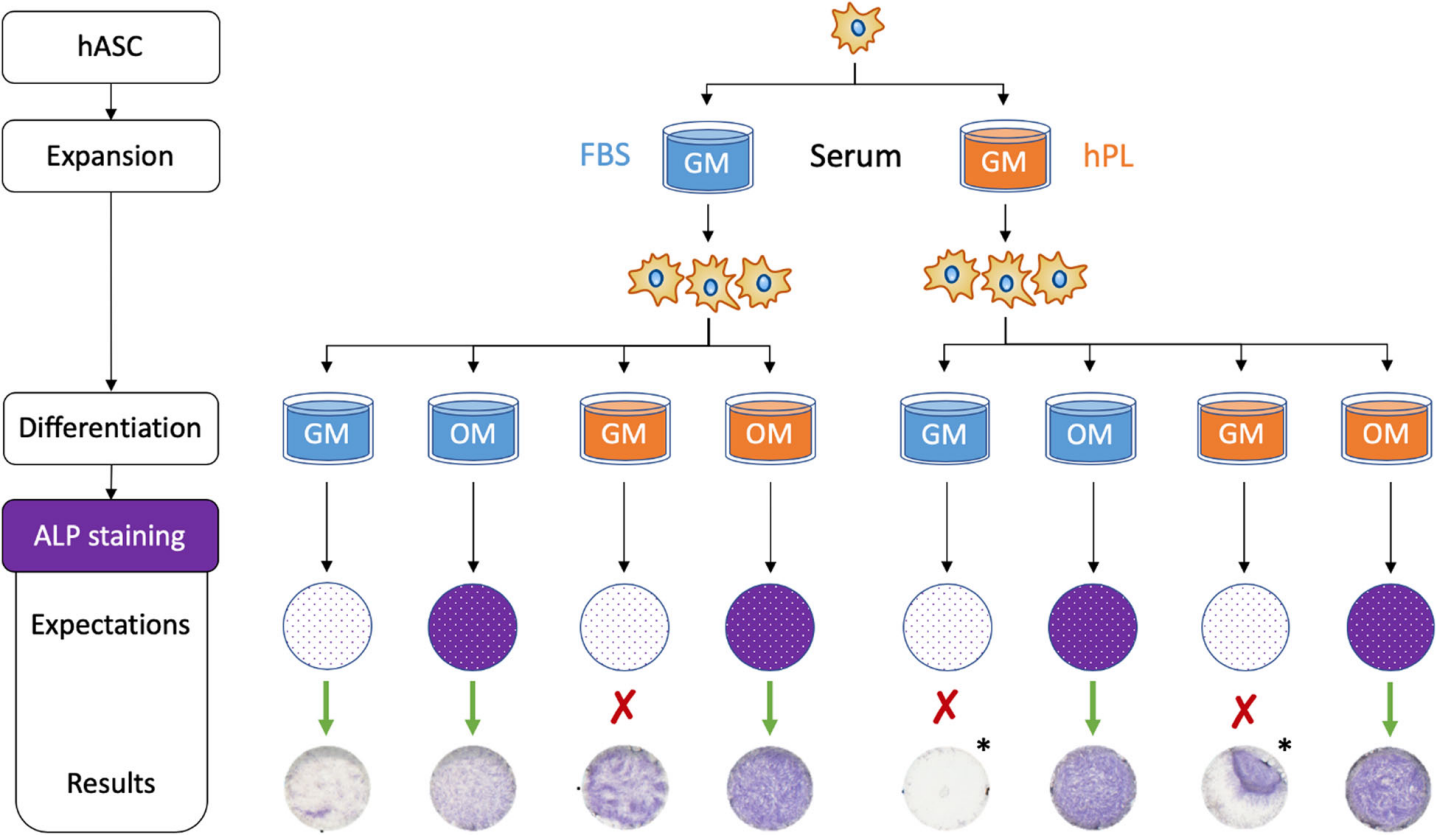
Pre-screening of the effect of the sera used during hASC expansion on the validity of controls in the subsequent osteogenic differentiation. hASC-EFS were expanded in either FBS- (blue) or hPL-based (orange) GM, followed by their differentiation in respective OM. For differentiation, both the expectations for the ALP staining and the actual staining results are shown. The latter are representative of 1 biological with 2 technical replicates for each condition. “*” denotes partial or complete cell layer detachment

To study whether the serum used during hASC expansion might inadvertently affect the subsequent osteogenic differentiation, hASC-EFS were expanded for 2 passages in either FBS- or hPL-based GM and then either maintained in these media or osteogenically induced in FBS- or hPL-based OM (Fig. 1). As expected, FBS-expanded hASC-EFS that were either maintained or osteogenically induced in FBS-based media confirmed the fidelity of both positive and negative controls with a sparse coloration in FBS-based GM and a strong coloration in FBS-based OM. When the same cells were maintained or osteogenically induced in hPL-based media, both controls showed an equally strong coloration, revealing a compromised negative control. hPL-expanded hASC-EFS that were maintained in FBS-based GM failed to validate the fidelity of the negative control as in both replicates the cell layer became completely detached prior to the ALP staining. A cell layer was similarly compromised when the same cells were maintained in hPL-based GM and the remaining adherent cells showed a strong coloration, thus invalidating the negative control. Both of the positive controls for hPL-expanded hASC-EFS, FBS- and hPL-based OM, showed strong colorations as expected. Thus, due to the false negative controls in hPL-expanded hASC-EFS, further DOE analyses were performed exclusively with FBS-expanded hASCs.

### hASC differentiation according to DOE conditions

**Fig. 2:**
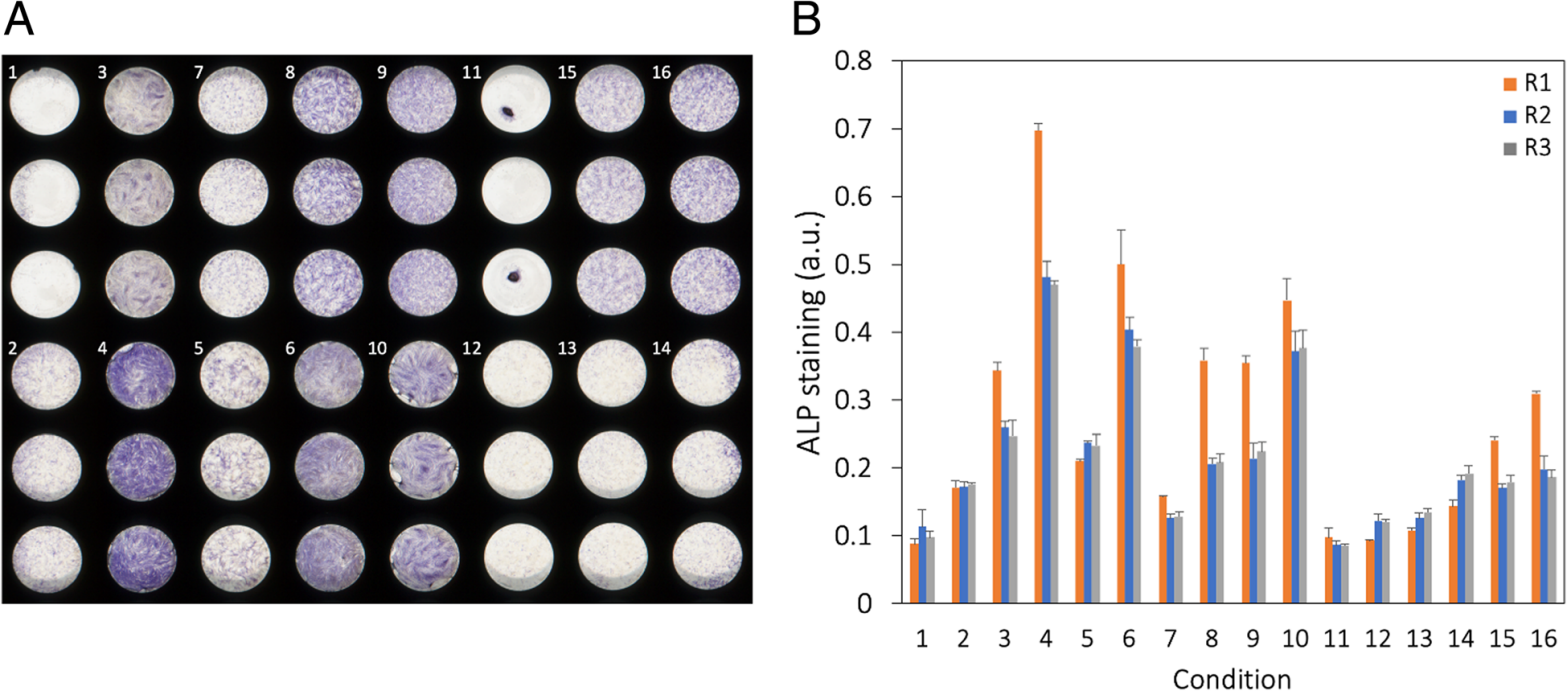
Results of the ALP staining and its quantification for all the conditions of the DOE analysis. **a** One of the three 96-well microplates after the ALP staining, displaying all 16 different DOE conditions with 3 technical replicates each. **b** Quantification of the ALP staining per condition per biological replicate (R1, R2, and R3) using absorbance detection. Results are expressed as means ± standard deviations of the 3 technical replicates

FBS-expanded hASC-ZB and hASC-EFS were seeded at a given density and induced with one of the corresponding induction media (Table 2) for a week before the ALP staining (Fig. 2a). Visually, there were marked differences between different conditions within the same biological replicate which was further confirmed by the quantification of the ALP staining using absorbance (Fig. 2b). While the absolute values for the quantified ALP staining differed between the biological replicates, the overall trends were consistent across all three. The average experimental error between them was calculated to equal 4.1%.

### Influence of hASC culture parameters on osteogenic differentiation via DOE analysis

**Fig. 3:**
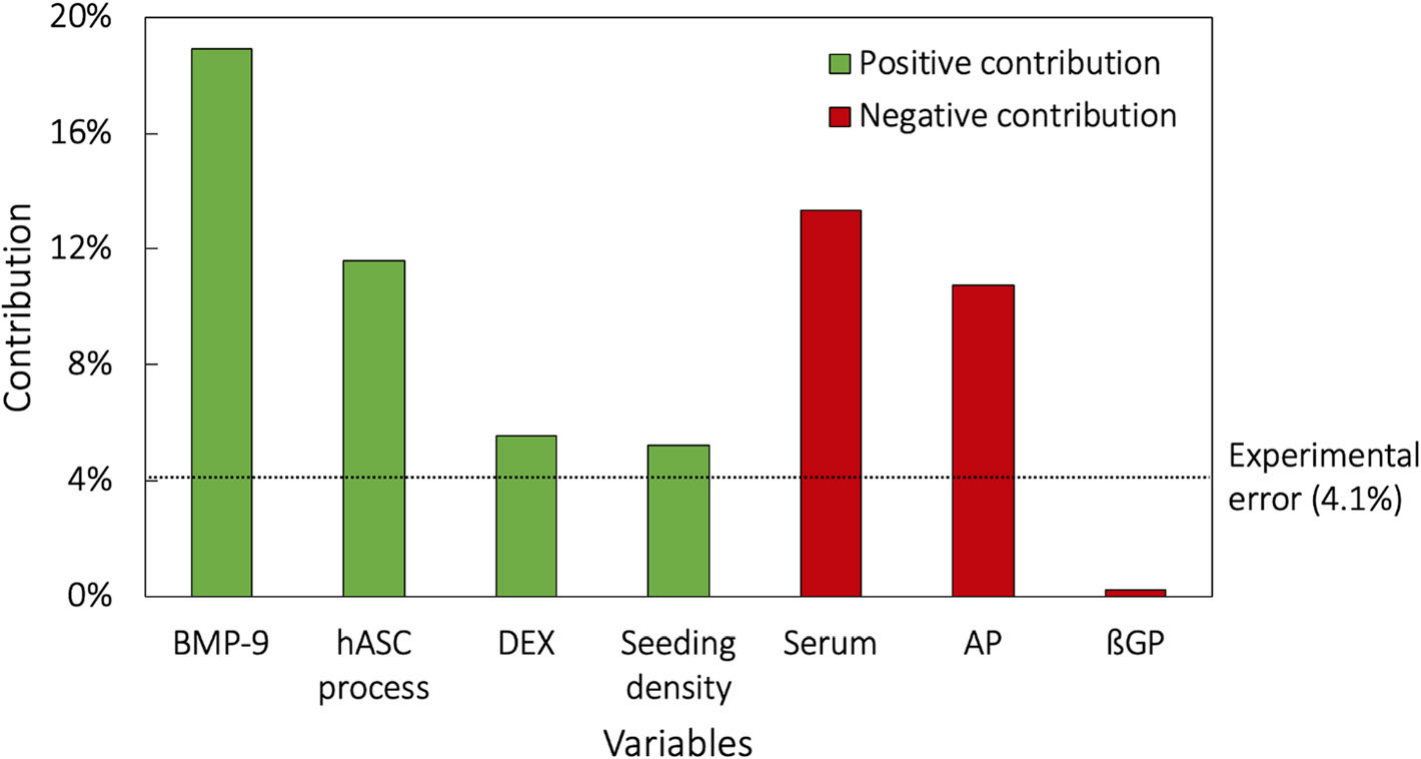
Calculated percent contributions of the variables toward the ALP expression. The variables had either a net positive or a net negative contribution to the ALP expression. If the (+) variability of the variable resulted in higher ALP expression, the contribution was positive; if it led to lower ALP expression, the contribution was deemed negative. The average experimental error between the three biological replicates (4.1%) was used as the significance limit, which resulted in the following ranking of the variables in terms of their final contribution: (1) BMP-9, (2) serum, (3) hASC source, (4) AP, (5) DEX, and (6) seeding density

The quantified results from the ALP staining (Fig. 2b) were encoded into Design-Expert® and analyzed to decode the relative contributions of the variables toward maximizing the ALP expression (Fig. 3). As mentioned previously, only single-variable contributions were considered. This is the main reason why the total sum of these contributions does not add up to 100% as intervariable interactions have not been accounted for. The analysis revealed that the variables had either a net positive or a net negative contribution toward maximizing the ALP expression. hASC source, seeding density, dexamethasone (DEX), and BMP-9 showed a net positive contribution, i.e., their (+) variability led to a higher ALP expression. On the other hand, the medium, serum, l-ascorbate-2-phospate (AP), and ßGP showed a net negative contribution, i.e., their (+) variability led to a lower ALP expression. To provide a statistically relevant ranking of these variables, the previously estimated experimental error between the biological replicates (4.1%) was used as the significance limit. In this case, the decreasing order of the contributions to the ALP expression was as follows: (1) BMP-9, (2) serum, (3) hASC source, (4) AP, (5) DEX, and (6) seeding density.

## Discussion

### Compromised negative controls for hASC differentiation due to hPL

To study whether hPL used during hASC expansion affects the eventual fidelity of both negative and positive controls for their osteogenic differentiation, hASC-EFS were expanded for 2 passages in either FBS- or hPL-based GM and then induced to undergo osteogenic differentiation. The ALP staining results point to the fact that the use of hPL, either during expansion or differentiation, likely results to compromised negative controls (Fig. 1). Even at a relatively low supplementation of 5%, hPL was able to induce spontaneous ALP expression of FBS-expanded hASCs maintained in hPL-based GM. While further assays and more biological replicates are needed to derive definitive conclusions, these results nevertheless provide further support to the previous studies that reported similar findings [8, 12, 13]. Surprisingly, human bone marrow mesenchymal stromal cells, cultured in a similar medium, did not show the false negative controls reported for hASCs, which highlight differences between these two cell types. In our study, the intensity of the coloration was visibly higher in hASC-EFS that came into contact with hPL as compared to those that were expanded and differentiated in FBS-based media. One likely explanation for the osteoinductivity of hPL is its composition. hPL, namely human platelet lysate, is obtained by lysing platelets found in human blood and is full of bioactive proteins and growth factors, including osteogenic ones such as BMPs and platelet-derived growth factors (PDGFs). While its precise composition is unknown, it may contain sufficient amounts of the latter to induce the osteogenic differentiation of hASCs. It may be interesting to further investigate the precise role of hPL on hASC cell differentiation, since hASC are currently explored in clinical trials as alternative to mesenchymal stromal cells. A full characterization including quantitative polymerase chain reaction (qPCR) of relevant marker genes like ALP, runt-related transcription factor 2 (RUNX2), and osterix (OSX) up to matrix mineralization may be needed prior to their use for in vivo assays.

### Confirmation of the differential importance of culture parameters via DOE analysis

Given that hPL-expanded hASC-EFS led to false negative controls, only FBS-expanded hASCs were used for the subsequent DOE analysis. Its results confirmed the high sensitivity of hASCs, namely the expression of ALP, toward different culture parameters. The intensity of the ALP staining varied widely and some conditions like n° 11 even led to the detachment of the cell layer (Fig. 2a). The quantified values of the ALP staining for each of the conditions (Fig. 2b) were then encoded within the generated table (Table 2) using Design-Expert®. While it allows many different modes of analysis, this work focused on the individual ranking of single variables for their effect in maximizing the ALP expression (Fig. 3). BMP-9 had the largest contribution to the ALP expression in hASCs, which confirmed its potential as an osteoinductive factor for their osteogenic differentiation. Previous studies have identified its role in adipogenesis [21] while others pointed to its potentially higher osteoinductivity to hASCs compared BMP-2 and BMP-7, which are more commonly applied for such a purpose. The results of this study corroborate the latter findings. The second most important variable for maximizing ALP expression was the serum. In accordance with the previously mentioned results (cf. Section “Compromised negative controls for hASC differentiation due to hPL”), hPL induced higher ALP expression than FBS, which is confirmed by the net negative contribution of this variable. hASC source was the third most important factor, with hASC-EFS [our (+) variability for this variable] contributing positively to higher ALP expression compared to hASC-ZB. It is difficult to make any definitive conclusions for why this is may be due to the fact that hPL is used in hASC-EFS isolation and processing protocols as the serum supplement, in large part to avoid the disadvantages of the FBS. hASCs may become pre-differentiated when processed and passaged in an hPL-based medium, thus leading to higher ALP expression. Interestingly, AP was the fourth important variable with its absence from the medium leading to a higher ALP expression. AP is an integral part of standard OM formulations [7], mainly responsible for the deposition of the extracellular matrix. Why its presence had an inhibitory effect on the extent of ALP expression is unknown but warrants a further study. DEX, another common OM component, was the fifth variable in terms of its influence. It is considered to be highly osteoinductive [22]; however, its effect on hASCs is varied as it makes part of the media used for their adipogenic differentiation as well [21]. This might explain why its contribution was lower than that of BMP-9. Finally, the seeding density was the sixth most important variable for maximizing ALP expression. This makes intuitive sense as higher seeding densities mean higher numbers of cells leading to higher amounts of ALP to stain in terms of its absolute quantities. While our analysis was focused on these several culture parameters, the application of DOE might be used to study also how hASC function depends on the known variabilities related to donor characteristics, collection method, and passage number [23].

## Conclusions

The DOE approach was applied to identify and rank important culture parameters that affect the ALP expression in order to optimize the osteogenic differentiation of hASCs. Our preliminary results show that, among the selected culture parameters, BMP-9, serum, AP, hASC source, DEX, and seeding density are important culture parameters that affect the osteogenic differentiation of hASCs. However, hPL may compromise hASCs stemness which merits a further in-depth investigation with qPCR for the expression of relevant genes and other established markers for multi-lineage differentiation. Thus, DOE is a versatile tool for the rapid pre-screening of culture parameters to identify the most important parameters in order to judiciously optimize culture conditions for a specific purpose. The combination of DOE with automated high-throughput screening methods such as high-content analysis could further improve the rapidity and fidelity of DOE analyses as they would allow to expand the analyzable parameter space and concurrently focus on several readouts.

## Additional file

Additional file 1: hASC phenotype, comparison of ALP staining and ALP enzymatic activity for the rapid measurement of the ALP expression and comparison of different ALP staining time points. (DOCX 2716 kb)

## Data Availability

The datasets used and/or analyzed during the current study are available from the corresponding author on reasonable request.

## Abbreviations

ALP: Alkaline phosphatase
AP: l-ascorbate-2-phospate
BMP: Bone morphogenetic protein
DEX: Dexamethasone
DMEM: Dulbecco’s modified Eagle’s medium
DOE: Design of experiments
FBS: Fetal bovine serum
GM: Growth medium
GMP: Good Manufacturing Practices
hASC: Human adipose-derived stromal cells
hASC-EFS: hASC supplied by Établissement Français du Sang (France)
hASC-ZB: hASC supplied by Zen-Bio, Inc. (USA)
hPL: Human platelet lysate
OM: Osteogenic medium
OSX: Osterix
PBS: Phosphate-buffered saline
PDGF: Platelet-derived growth factor
qPCR: Quantitative polymerase chain reaction
RUNX2: Runt-related transcription factor 2
ßGP: ß-glycerophosphate
