# Supplementary material for: Design of experiments to assess the effect of culture parameters on the osteogenic differentiation of human adipose stromal cells
Source: Stem Cell Res Ther. 2019 Aug 14;10:256. doi: 10.1186/s13287-019-1333-7 (PMC6694725; doi:10.1186/s13287-019-1333-7)
Supplement: Supplementary file 1 — hASC phenotype, comparison of ALP staining and ALP enzymatic activity for the rapid measurement of the ALP expression and comparison of different ALP staining time points. (DOCX 2716 kb) [file 13287_2019_1333_MOESM1_ESM.docx]

**Design of Experiments to assess the effect of culture parameters on the osteogenic differentiation of human adipose stromal cells**

Mirasbek Kuterbekov^a,b,c^, Paul Machillot^b,c^, Francis Baillet^d^, Alain M. Jonas^a^, Karine Glinel^a^, Catherine Picart^b,c,^*

^a^ Université catholique de Louvain, Institute of Condensed Matter & Nanosciences (Bio & Soft Matter), Croix du Sud 1, box L7.04.02, 1348 Louvain-la-Neuve, Belgium

^b^ CNRS, LMGP, 3 parvis Louis Néel, 38016 Grenoble, France

^c^ Grenoble Institute of Technology, University Grenoble Alpes, LMGP, 3 parvis Louis Néel, 38016 Grenoble, France

^d^ Université Grenoble Alpes, CNRS, Grenoble INP, SIMAP, 1130 rue de la Piscine, 38402 Saint-Martin d'Hères, France

* corresponding author: [catherine.picart@grenoble-inp.fr](mailto:catherine.picart@grenoble-inp.fr)

# Supplementary information

## 1. Reported phenotypes for hASC-ZB and hASC-EFS

| Cell marker | CD13 | CD14 | CD19 | CD29 | CD31 | CD34 | CD44 | CD45 | CD73 | CD90 | CD105 | CD133 |
| --- | --- | --- | --- | --- | --- | --- | --- | --- | --- | --- | --- | --- |
| hASC-ZB | N/I | - | N/I | + | - | - | + | - | N/I | N/I | + | - |
| hASC-EFS | + | N/I | - | N/I | - | - | + | - | + | + | + | N/I |

N/I: no information supplied.

## 2. Comparison of ALP staining and ALP enzymatic activity for the rapid measurement of the ALP expression

Both ALP staining and ALP enzymatic activity methods can be used to quantify the ALP expression in hASCs. To allow for the rapid assessment of different culture parameters, both ease and fidelity of the measurements are important. To analyze their suitability for our particular application, hASC-EFS pre-cultured in FBS-based growth medium were osteogenically induced using the corresponding media from the Hadamard matrix generated from **Table 2** (main manuscript) and analyzed for the ALP expression using either the previously described ALP staining (1) or ALP enzymatic activity methods (2). For the latter, hASC-EFSs were washed twice with PBS (200 µL/well) and lysed with Triton-X100 (0.5 % in PBS, 100 µL/well). 20 µL of the supernatant was mixed with a buffer (pH 10) containing 0.1 M 2-amino-2-methyl-1-propanol, 1 mM magnesium chloride and 9 mM *p*-nitrophenyl phosphate (pNPP, Euromedex) in a 96-well cell culture microplate (Greiner Bio-One). The reaction was followed for 10 min by measuring the absorbance every 30 s at 405 nm using a microplate reader (TECAN Infinite M1000). The ALP enzymatic activity was expressed as µmol of pNP/min/mg of protein. The total protein content of the samples was determined using a BCA kit for protein determination (Bicinchoninic Acid Protein Assay Kit), according to the manufacturer’s instructions.

The results of the analysis show that the ALP staining generated more reproducible readings as judged by the standard deviation (error bars) between technical replicates (**Figure S1A**). The ALP enzymatic activity showed much higher variations across more replicates (**Figure S1B**). This is likely linked to the small working volumes used to estimate both the kinetics of the ALP reaction and the total amount of protein inside each well. These discrepancies due to the insufficient sensitivity of the ALP enzymatic activity method might also explain the contradictions in the measurement of the ALP expression for the same DOE conditions between the two studied methods.


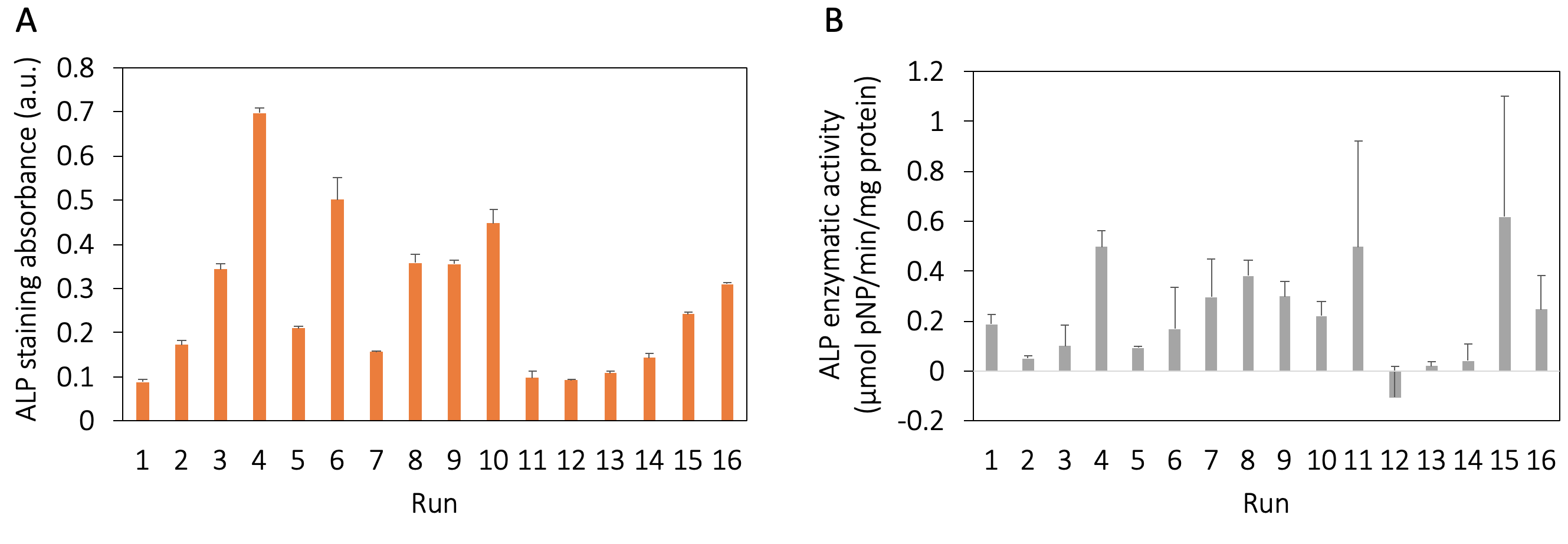


**FIGURE S1.** **Graphs of the ALP expression in hASCs osteogenically induced according to the DOE conditions (*cf.* Table 2) and quantified using either (A) ALP staining or (B) ALP enzymatic activity**. The results of ALP staining show comparatively smaller variations (error bars) between replicates compared to the results of ALP enzymatic activity. Results are expressed as means ± standard deviations of three technical replicates.

## 3. Comparison of different ALP staining time points

Different time points for ALP staining were carried out to determine the shortest, thus fastest, duration needed to obtain reliable results. For this purpose, ALP staining was carried at day 7 (D7), day 10 (D10) and day 14 (D14) on hASC-ZB and hASC-EFS that were osteogenically induced in four different media:

1. Growth media (GM): DMEM/F-12 + 10 % FBS + 1 % penicillin/streptomycin.
2. Supplemented medium 1 (SM-1): GM + 100 µM L-ascorbate-2-phosphate + 10 mM ß-glycerophosphate.
3. Supplemented medium 2 (SM-2): GM + 250 µM L-ascorbate-2-phosphate + 10 mM ß-glycerophosphate.
4. Osteogenic medium (OM): SM-1 + 100 nM dexamethasone.

The results of the staining for hASC-ZB (**Figure S2A**) and hASC-EFS (**Figure S2B**) showed that differences in ALP staining among different media were already visible at D7. At D10 and D14, cell layer detachment was observed for both hASC-ZB and hASC-EFS. The quantification of the ALP staining showed that it was generally higher at D7 compared to D10 and D14.


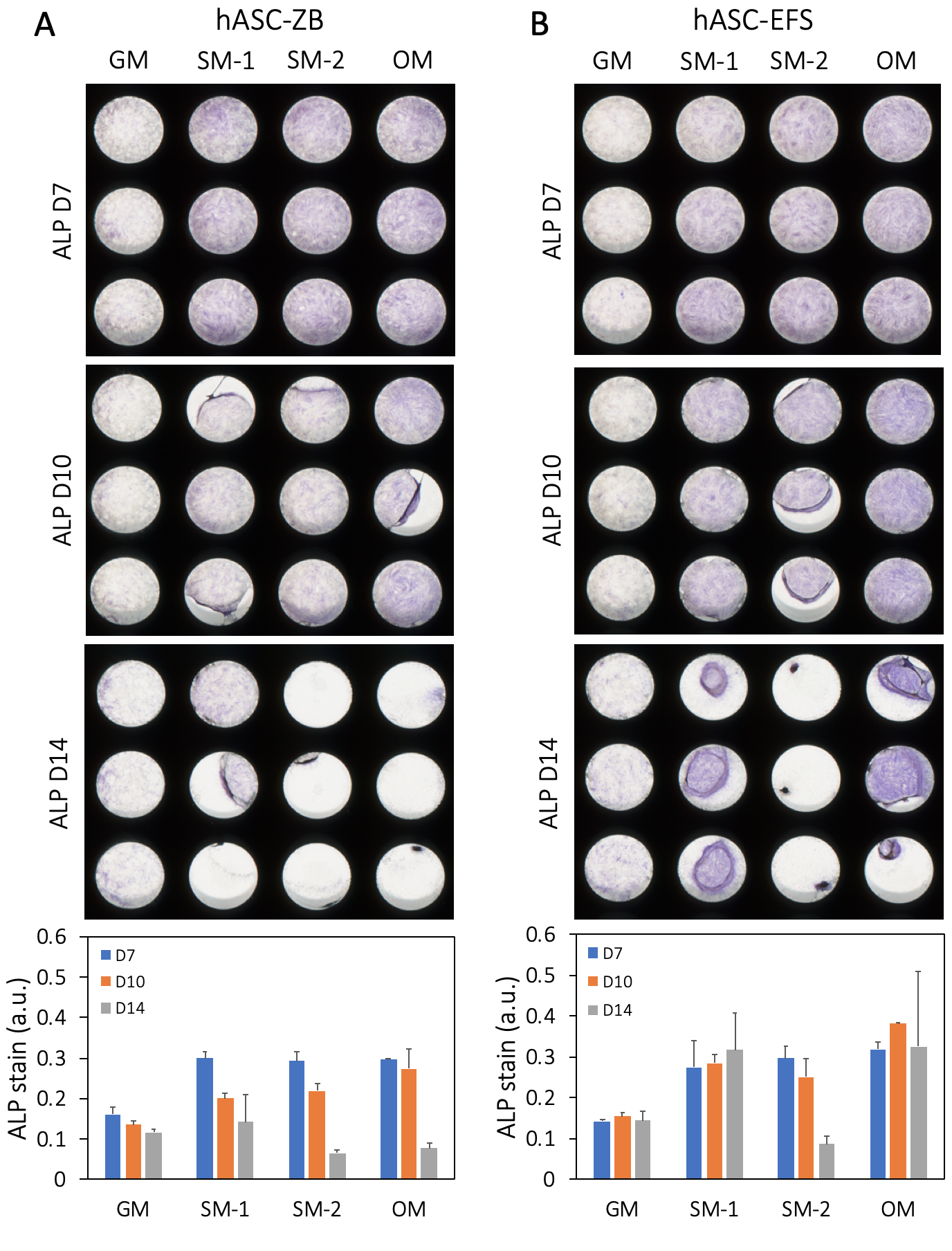


**FIGURE S2.** **The ALP expression in (A) hASC-ZB and (B) hASC-EFS at different time points.** The ALP expression was visualized and quantified for GM, SM-1, SM-2 and OM at D7, D10 and D14. Cell layer detachment was observed for D10 and D14 for both hASC-ZB and hASC-EFS, but not at D7.

# References

1. Crouzier T, Ren K, Nicolas C, Roy C, Picart C. Layer-by-layer films as a biomimetic reservoir for rhBMP-2 delivery: controlled differentiation of myoblasts to osteoblasts. Small. 2009 Mar;5(5):598–608.

2. Kuterbekov M, Machillot P, Lhuissier P, Picart C, Jonas AM, Glinel K. Solvent-free preparation of porous poly(l-lactide) microcarriers for cell culture. Acta Biomater. 2018 Jun 6;75:300–11.
